# Texas State Medical Convention

**Published:** 1919-04

**Authors:** 


### TEXAS STATE MEDICAL CONVENTION, WACO, TEXAS, MAY 13-15.

### Announcements—Business.

The Registration Office will be situated in the Chamber of Commerce, Fifth and Washington Streets; here badges and programs will be given out. Members are urged to register as soon as they arrive in Waco.

An Information Bureau will be conducted in connection with the Registration Office, where all necessary information may be received concerning hotels, places of meeting, railroads, street cars, entertainments, etc.

Scientific Sessions will be held in the following halls:

Hall No. 1.—Auditorium, Sixth and Columbus Streets.

Hall No. 2.—Knights of Columbus Hall, Sixth and Columbus Streets.

Hall No. 3.—Y. M. C. A. Building, Sixth and Washington Streets.

Hall No. 4.—Parrish House, Sixth and Columbus Streets.

The House of Delegates meets in the Chamber of Commerce Hall, Fifth and Washington Streets.

The Opening Exercises and all of the General Sessions meet in Hall No. 1, the Auditorium, Sixth and Columbus Streets.

All mail and telegrams should be directed to the Information Bureau, care of the State Medical Association of Texas, Chamber of Commerce Building, Waco, Texas.

Members are urged to leave their names and local addresses at the Information Bureau at the time of registration, in order that mail and telegrams may be promptly delivered.

Official announcements will be posted on the Bulletin Board at the Information Bureau.

Those desiring reservations for exhibition space should apply to Dr. H. M. Lanham, Chairman Exhibit Committee, Waco.

### Social.

At the Hotels Raleigh, Metropole, Waco, State House and at the Chamber of Commerce, a reception committee will be present, representatives of the Woman's Auxiliary of the McLennan County Medical Society, to receive visiting women until noon Wednesday, and to furnish any information desired.

TUESDAY, MAY 13TH.

5:00 to 7:00 p. m. in the Ball Room of the Hotel Raleigh—

Tea-Musicale, for the introduction and acquaintance of guests.

7:45 p. m.—Assembling in the lobby of the Raleigh for a visit to the Hippodrome.

WEDNESDAY, MAY 14TH.

9:15 a. m.—Automobiles will leave the Hotel Raleigh for the Waco Boating and Fishing Club, where the Woman's Auxiliary will give a swim and breakfast. Mrs. E. H. Cary, President of the Woman's Auxiliary of the State Medical Association, will be the guest of honor. Following the breakfast, there will be held in the Boating and Fishing Club Building a business meeting of the State Woman's Auxiliary.

6:00 to 8:00 p. m.—Mrs. W. O. Wilkes, 1608 Washington Street, at home, complimentary to visiting women.

9:00 p. m.—President's reception on the second floor and ball room of the Hotel Raleigh.

THURSDAY, MAY 15TH.

9:00 a. m.—Drive over the city for visiting ladies.

**Alumni Banquets.**

The Committee on Arrangements this year contemplates no other functions on Tuesday night except Alumni Banquets, the Banquet of the Ex-Presidents of the State Association and such other personal and individual functions as may be desired. For arrangements, address Dr. C. E. Collins, Chairman, Alumni Banquet Committee, Waco.

### RATES

It is doubtful if any reduced rates to the State Meeting can be secured this year.

**Hotels.**

| | |
|---|---|
| *Raleigh Hotel*—$1.25 up | (250) |
| *State House*—$1.50 to $2.50 | (75) |
| *New Exchange*—$1.00 | (150) |
| *Waco*—$1.25 to $3.00 | (70) |
| *Savoy New*—$1.00 to $3.00 | (45) |
| *Natatorium*—$1.00 up | (50) |
| *St. Charles*—$1.00 to $1.50 | (75) |
| *Terminal*—$1.00 to $1.50 | (250) |
| *Brazos*—$1.00 up | (100) |
| *New Katy*—$1.00 to $1.50 | (100) |
| *Metropole*—$1.00 up | (150) |
| *Teitz*—$1.00 up | (150) |

### FIRST DAY, TUESDAY, MAY 13TH.

**General Session—Opening Exercises.**

**10:30 a. m., Hall No. 1.**

**Auditorium**

**Sixth and Columbus Streets.**

*Invocation* .......... Rev. R. E. Goodrich
*Welcome Address on Behalf of Waco*......Mayor Ed. McCullough
*Welcome Address on Behalf of McLennan County Medical Society*..........Dr. W. O. Wilkes, President
*Response and President's Annual Address*.......... ..........Dr. S. P. Rice, Marlin
*Benediction* .......... Dr. W. P. Whitsell

**Afternoon Sessions.**

The section on Surgery will meet from 2:00 to 6:00 at the Auditorium; the section on Medicine and Diseases of Children from 2:00 to 6:00 at Knights of Columbus Hall, Sixth and Columbus Streets; and the section on State Medicine and Public Hygiene at the Y. M. C. A., at Sixth and Washington.

Second Day, May 14th.

The section on Surgery will continue its session from 9:00 a. m. to 4. p. m. at the Auditorium; the section on Medicine and Diseases of Children at the same hours at the Knights of Columbus Hall; the section on State Medicine and Public Hygiene from 9:00 to 12:00 at the Y. M. C. A.; the section on Gynecology and Obstetrics from 1:30 p. m. to 4 p. m. at the Y. M. C. A.; the section on Opthalmology, Otology, Laryngology and Rhinology from 9:00 a m. to 4:00 p. m. at Parish House; and the General Session (Joint Meetings of all Scientific Sections) from 4:00 to 6:00 p. m., at the Auditorium. The President's Reception will be at 9:00 p. m. at the Hotel Raleigh.

Third Day, Thursday, May 15th.

The section on Surgery will continue its sessions from 9:00 a. m. to 5:00 p. m. at the Auditorium; the section on Medicine and Diseases of Children will continue its sessions from 9:00 a. m. to 5:00 p. m. at Knights of Columbus Hall; the section on Gynecology and Obstetrics will continue its sessions from 9:00 a. m. to 5:00 p. m. at the Y. M. C. A.; and the General Session will be held at the Auditorium from 5:00 to 6:00 p. m., concluding with the introduction of newly elected officers.

